# Effects of *Rhizophagus irregularis* on Photosynthesis and Antioxidative Enzymatic System in *Robinia pseudoacacia* L. under Drought Stress

**DOI:** 10.3389/fpls.2017.00183

**Published:** 2017-02-16

**Authors:** Fei He, Min Sheng, Ming Tang

**Affiliations:** ^1^College of Forestry and Landscape Architecture, South China Agricultural UniversityGuangzhou, China; ^2^College of Forestry, Northwest A&F UniversityYangling, China; ^3^School of Modern Agriculture and Biotechnology, Ankang UniversityAnkang, China

**Keywords:** arbuscular mycorrhizal fungi, black locust, reactive oxygen species, antioxidant enzyme, gene expression

## Abstract

Arbuscular mycorrhizal (AM) fungi colonize roots improving plant water status and tolerance to drought. However, it is not clear whether the presence of AM would affect the photosynthesis and antioxidant gene-enzymes response, which help to alleviate drought stress of the host plant. Here, pot experiments were performed to investigate the effects of *Rhizophagus irregularis*, an AM fungus, on the tissue water content, photosynthesis, reactive oxygen species (ROS) production, antioxidant enzyme activity and gene expression in black locust (*Robinia pseudoacacia* L.) seedlings which were subjected to well-watered or moderate drought stress. Mycorrhizal symbiosis increased relative water content (RWC) of plant roots and leaves, promoted the accumulation of biomass and chlorophyll (Chl) content, and improved photochemistry efficiency, regardless of watering regimes. Mycorrhizal plants had higher SOD, POD, CAT, APX, and GR activities, and the transcript levels of *Cu/Zn-SOD. APX* and *GR*, but lower O_2_^-^, H_2_O_2_ and MDA concentrations in leaves and roots of black locust under drought and well-watered conditions. Results from the present study indicate that AM fungus (*R. irregularis*) symbiosis can enhance photosynthesis and ROS scavenging capabilities and increase RWC of leaves and roots to alleviate drought stress in black locust. Further research is needed to elucidate the relations among AM fungi and the metabolic pathways of antioxidant enzymes, and the function of antioxidant genes regulated by mycorrhizal symbiosis with the purpose of revealing the mechanisms of mycorrhizal-induced plant tolerance to drought stress.

## Introduction

Drought is a kind of the most common abiotic stress affecting plant growth and development (Wu et al., 2013a). DS interrupts the balance of ROS (e.g., superoxide and H_2_O_2_) in plants, which are harmful to lipids, proteins, and plant cell tissues. Under DS, excessive formation of ROS can lead to oxidative damage in plants through lipid peroxidation, protein oxidation, and other mechanisms (Sharma et al., 2012). In order to avoid oxidative damage, plant has developed a complicated antioxidant protective system that scavenges excess ROS (Li and Yi, 2012). Multiple antioxidant enzymes [e.g., superoxide dismutase (SOD); peroxidase (POD); catalase (CAT); ascorbate peroxidase (APX); and glutathione reductase (GR)], are involved in plant response to DS, and the production of antioxidant enzymes is considered as a protection mechanism of plant against DS (Li and Yi, 2012). Plant responses to DS also involve other numerous mechanisms, as well as various regulatory pathways, and drought-associated genes, such as these related with signaling pathway or these enzymes which synthesize structural or functional metabolic products (Gruber et al., 2009). In particular, the responses of antioxidant enzymes or transcripts are important for studying plant stress responses (Becana et al., 2010). However, enzyme activities reflect variations in entire capacity averaged over distinct isoforms and compartments; transcript analysis allows the difference of gene-specific responses (Bian and Jiang, 2009). As a result, it is unclear if a consistent view is available in the antioxidant enzyme activities and transcript regulation under drought.

Arbuscular mycorrhizal fungi form symbiotic relationships with most terrestrial plant species (Smith and Read, 2008). AM symbiosis can protect host plants against the detrimental effects of DS (Bárzana et al., 2012). Several mechanisms have been proposed to explain the protective role of AM symbiosis in host plants against DS. For instance, a direct hyphal pathway for the transport of water from soil to plants (Querejeta et al., 2012), changes of soil water retention characteristics (Daynes et al., 2013), enhancement of water use efficiency for plants (Bárzana et al., 2012), and increasment of antioxidant enzyme activities against oxidative injury (Fouad et al., 2014). The last has been considered as the most important mechanism of AM symbiosis, as many reactions related to environmental stresses (e.g., DS) result in the generation of ROS and induce oxidative injury in plants (Ruiz-Lozano, 2003).

Arbuscular mycorrhizal symbiotic development leads to the formation of arbuscule structures within the host plant cells, which are thought to be the major locus of signal and nutrient exchange among the host and its fungal partners (Etemadi et al., 2014). The cumulation of H_2_O_2_, as well as the transcript levels of *CAT* and *POD* have been detected in plant cells containing arbuscules (Lambais et al., 2003; Fester and Hause, 2005). Previous studies have shown that a gene encoding Cu/Zn-SOD has been reported in the mycorrhizal fungus *Gigaspora margarita* (Lanfranco et al., 2005). Still, little is known about the antioxidant gene (e.g., *APX. GR*) responses on the host plant partner, especially during DS.

Black locust (*Robinia pseudoacacia* Linn.), a kind of fast-growing legume tree species, originated in North America and was cultivated widely on eroded or poor soils (Tian et al., 2003). Black locust trees have high economic values and a huge potential use in pharmaceuticals, garden, and bioenergy industries (González-García et al., 2011). It plays a positive role in increasing soil available P pool (Wang et al., 2012), promoting soil organic carbon sequestration (Yüksek, 2012), and improving soil quality in arid and semi-arid regions (Xu et al., 2014). AM fungi can form the structure of mutualistic symbiosis with black locust and remarkably improve drought resistance in the host plant (Yang et al., 2014). Previous research demonstrated that AM fungi are able to enhance drought-resistance of black locust seedlings by improvement of water use efficiency, photosynthesis, and nutrient uptake (Yang et al., 2014). In spite of this, concerning the symbiosis of host and their fungal partners, the mechanisms of drought response are extremely complex owing to the interaction effect between host and their partners.

Here, we wanted to know whether the presence of AM would affect the photosynthesis and antioxidant gene-enzymes responses of black locust in this study. We hypothesize that under DS, AM fungal colonization of black locust assists in the photosynthesis and antioxidant gene-enzymes response, which help to alleviate DS of the host plant. To test this hypothesis, we examined the changes of photosynthesis, antioxidant enzyme activity and gene expression in AM and NM black locust plants under DS and WW.

## Materials and Methods

### Plant, Soil Preparation, and AM Inoculum

Black locust seeds were purchased from a local market (Yangling, China). Seeds were surface disinfected with 10% NaClO for 10 min. After washes with sterile water eight times, seeds were pre-germinated by laying on moist filter paper in 9-mm diameter Petri dishes (30 seeds⋅dish^-1^) at 25°C. Ten-day-old seedlings were transplanted into plastic pots (top diameter 100 mm; depth 80 mm), with three seedlings per pot. Fifteen days later, plants of uniform growth were kept singly per pot.

Soil sample was collected down to a depth of 0–20 cm (5 cm of soil surface removal) in a field (Yangling City, China). The soil contained 13.78 P mg/kg, 33.98 N mg/kg, 152.5 K mg/kg, and 16.2 g/kg organic matter, with pH 7.6 (1:5, *w*/*v*, soil/water). After air-dried, the soil was sieved with a 2 mm mesh. Then it was mixed with fine sand (1:1, *v*/*v*) and treated by moist-heat sterilization for 1.5 h at 121°C, to obtain the growth substrate.

The AM fungus species, *Rhizophagus irregularis* (N. C. Schenck and G. S. Smith) C. Walker and A. Schüßler (No. BGCBJ09) (synonym for *Glomus intraradices* DAOM 197198) was purchased from Beijing Academy of Agriculture and Forestry Sciences (China). The AM inoculum comprised a mixture of mycelia, root segments, spores, and soil-sand.

### Experimental Design

Pot experiments were conducted from March to June in 2014. Black locust was grown in a pot containing 0.55 kg of growth substrate each, and placed in the greenhouse at 25–33°C with 15 h light period. The experiment was a 2 × 2 complete factorial design that consisted of two inoculation treatments and two soil watering regimes. Treatments included two factors: (i) The AM fungal inoculation with *R. irregularis* (AM) or the non-mycorrhizal control (NM); (ii) The soil water regime, either DS or WW. In total, each seedling was received 10 g of inoculum (*n* = 100); and the other part of seedlings (controls; *n* = 100) were inoculated with 10 g of autoclaved inoculum, plus 10 mL of inoculum filtrate to get rid of AM propagules.

Two hundred pots were distributed in a randomized complete block design, with fifty replicates of one seedling per treatment. All seedlings were well-watered and maintained at 75–80% of field water-holding capacity at first. After 75 days, the seedlings including 100 AM and 100 NM treatment were each randomly split up into two groups of 50. Then 50 AM and 50 NM seedlings that were to be suffered from DS were left unwatered until the soil reached 35–40% of field water-holding capacity. The remaining AM (*n* = 50) and NM (*n* = 50) seedlings received well-watered and were kept 75–80% of field water-holding capacity. All seedlings were maintained at the set field water-holding capacity for 14 days. Throughout the experimental period, all seedlings were weighed and supplied water each day at 17:00 to keep the designated field water-holding capacity.

At the end of the experiment, six replicates of each treatment were randomly selected to measure Chl fluorescence parameters, and then harvested to determine dry weight, photosynthetic pigment concentration, and mycorrhizal colonization. Six plants were randomly selected to assay ROS production, lipid peroxidation, and antioxidant enzyme activity. Three plants were frozen in liquid nitrogen and stored at -80°C for gene expression analysis.

### Growth Measurement and Mycorrhizal Colonization Analysis

Roots, stems, and leaves were harvested separately and washed free from soil with running tap water, and fresh weights (FWs) of tissues were recorded. Mycorrhizal colonization was measured using the method of Phillips and Hayman (1970) with minor modifications. Fresh roots were gently washed with running tap water and cut into 1-cm pieces. Tissue specimens were cleared with 10% KOH at 90°C for 30 min, bleached with alkalized H_2_O_2_ for 20 min, acidified with 1% HCl at room temperature for 30 min, stained with trypan blue solution (500 mL glycerol, 450 mL water, 50 mL % HCl, and 0.05% trypan blue) at 90°C for 5 min, and finally destained with lactic acid-glycerin (1:1, v:v) at room temperature. Mycorrhizal colonization was estimated by using the grid line intersection method under a light microscope (Giovannetti and Mosse, 1980). Arbuscular, vesicular, hyphal and total root colonizations were defined as the percentage of root segments colonized for fine root sample, respectively.

### Measurement of Hydraulic Parameters

For relative water content (RWC) determination, and the FW, including leaf fresh weight (LFW), stem fresh weight (SFW), and root fresh weight (RFW), was estimated immediately after collection. The turgid weight (TW) was measured by soaking the tissue samples in distilled water for 24 h, then we measured the total dry weight (DW), leaf dry weight (LDW), stem dry weight (SDW) and root dry weight (RDW) of tissues through drying in a hot air oven (80°C) at constant weight. RWC, aboveground DW and total DW were calculated according to the following formula:

$$\begin{array}{c} \text{RWC}(\%)=[(\text{FW-DW})/\left(\text{TW-DW}\right)]\times 100\\ \text{Aboveground dry weight}(\text{g per plant})=\text{SDW+LDW}\\ \text{Total dry weight}(\text{g per plant})=\text{RDW+SDW+LDW} \end{array}$$

### Measurement of Leaf Pigment

The Chl *a*, Chl *b* and carotenoid (Car) concentration was determined by using the method of Gao (2000). Fresh leaves (the sixth leaf from the top down) were washed free from contamination with deionized water, and fresh leaves (100 mg) were homogenized in 25 mL 80% acetone in darkness for 12 h at 25°C. The concentration of photosynthetic pigment was estimated by using a spectrophotometer (UV-1800, Shimadzu, Japan) at 663, 646, and 470 nm. The Chl and Car concentration was calculated as follows:

$$\begin{array}{c} Chl\,a(mg\,g^{-1}\,FW)=(12.21\times A_{663}-2.81\times A_{646})/FW\\ Chl\,b(mg\,g^{-1}\,FW)=(20.13\times A_{646}-5.03\times A_{663})/FW\\ Car(mg\,g^{-1}\,FW)=\frac{1000\times A_{470}-3.27\times Chl\,a-104\times Chl\,\;b}{229\times FW} \end{array}$$

Where A_λ_ = absorbance at λ (nm); FW, leaf fresh weight.

### Measurement of Chl Fluorescence Parameters

To measure Chl fluorescence parameters, six black locust seedlings of each treatment were dark-adapted for 30 min, then Chl fluorescence parameters were directly measured on the fifth youngest leaf of each seedling by using a Chl fluorometer (MINI-Imaging-PAM, Walz, Germany). The minimal fluorescence (F_0_) for dark-adapted leaf was recorded firstly. Then, a saturating pulse of 2,000 μM (photon) m^-2^ s^-1^ was made for 3 s to record the maximal fluorescence (F_m_) for dark-adapted leaf (Gong et al., 2013). Next, the leaves were placed under actinic light of 300 μM m^-2^ s^-1^ to determine F_m_′ (the maximum of fluorescence yield for light-adapted leaf), F_0_′ (the minimum of fluorescence yield for light-adapted leaf), and F_s_ (the steady-state value of fluorescence yield for light-adapted leaf). The maximal quantum yield of PSII for dark-adapted leaf (F_v_/F_m_), the actual photochemical yield of PSII (Φ_PSII_), the photochemical quenching (q_p_), as well as non-photochemical quenching coefficient (NPQ) were determined by using the following formula (Zai et al., 2012):

$$\begin{array}{c} q_{p}=\frac{{F_{m}}^{\prime }-F_{s}}{{F_{m}}^{\prime }-{F_{0}}^{\prime }};\Phi_{PSII}=\left({F_{m}}^{\prime }-F_{s}\right)/{F_{m}}^{\prime };\\ F_{v}/F_{m}=(F_{m}-F_{0})/F_{m};\text{NPQ=}(F_{m}-{F_{m}}^{\prime })/{F_{m}}^{\prime } \end{array}$$

### ROS Production and Lipid Peroxidation Analysis

Reactive oxygen species production and lipid peroxidation in plant tissues were analyzed by spectrophotometry. Root and leaf O_2_^-^ concentrations were determined by using the method of Zou et al. (2014) with minor modifications. Briefly, fresh tissues (0.2 g) were homogenized in 5 mL of 65 mM phosphate buffer solution (pH 7.8) at 4°C and centrifuged at 10,000 × *g* for 10 min. The extract supernatant (1 mL) was mixed with 0.9 mL of 65 mM phosphate buffer solution (pH 7.8), and 0.1 mL of 10 mM hydroxylamine hydrochloride in a water bath at 25°C for 30 min. Finally, this mixture (1 mL) was mixed with 1 mL of 17 mM *p*-aminobenzene sulfonic acid, and 1 mL of 7 mM α-naphthylamine and kept in a water bath at 25°C for 20 min, followed by determination of the absorbance at 530 nm. The content of O_2_^-^ was expressed as nM g^-1^ DW.

Hydrogen peroxide concentration was determined as described by Velikova et al. (2000) with minor modifications. Fresh tissues (0.5 g) were ground in 5 mL of 0.1% TCA at 4°C and centrifuged at 10,000 × *g* for 20 min. The extract supernatant (1 mL) was added to 1 mL of 10 mM potassium phosphate buffer (pH 7.0), and 2 mL of 1 M KI. The absorbency of the reaction mixture was measured at 390 nm, with a H_2_O_2_ standard curve. The H_2_O_2_ concentration was expressed as mM g^-1^ DW.

Determination of MDA concentration was performed according to the method of Kramer et al. (1991) with minor modifications. Fresh tissues (0.5 g) were homogenized in 5 mL of 0.1% TCA at 4°C and centrifuged at 12,000 × *g* for 10 min. Next, 1 mL of the supernatant was mixed with 4 mL of 0.5% thiobarbituric acid in 20% TCA. Samples were heated at 90°C for 30 min in a water bath and the reaction was stopped in ice bath. Centrifugation was performed at 12,000 × *g* for 10 min, followed by determination of absorbance at 532 and 600 nm. The concentration of MDA was expressed as mM MDA g^-1^ DW.

### Antioxidant Enzyme Activity Analysis

Frozen plant sample (approximately 0.5 g) was thawed and digested by 5 mL of phosphate buffer solution (50 mM; pH 7.8) for 10 min. Then extract was centrifuged at 6,500 × *g* at 4°C for 15 min and the supernatant was collected for the estimation of SOD and CAT activity.

The SOD activity was estimated as described by Giannopolitis and Ries (1977) with minor modifications. The reaction mixture was comprised of 50 μL of enzyme extract, phosphate buffer (50 mM; pH 7.8), 130 mM methionine, 20 μM riboflavin, 100 μM EDTA-Na_2_ and 75 μM NBT. The absorbance was measured at 560 nm, and SOD activity per unit was expressed as the enzyme amount that inhibits the reduction of nitroblue tetrazolium by 50%.

For estimation of CAT activity, the assay mixture contained enzymatic extract and H_2_O_2_ (0.1 M) for 2.5 mL each, incubated for 10 min at 30°C, and added 2.5 mL of 10% H_2_SO_4_. Then 0.1 M KMnO_4_ was used for titrating that mixture to pink color (Goldblith and Proctor, 1950). CAT activity was estimated through recording the change of absorbance for 1 min at 240 nm, and a unit of CAT activity was expressed as the enzyme amount that catalyzing the breakdown of H_2_O_2_ (1 μM) during 1 min.

The POD activity was estimated through measuring the absorbance of a mixture comprising 25 mM phosphate buffer, 0.05% guaiacol, 10 mM H_2_O_2_, and enzyme extract at 470 nm (Hemeda and Klein, 1990).

The APX activity was determined as described by Nakano and Asada (1981) with minor modifications. Assay mixture was comprise of phosphate buffer (50 mM; pH 6.0), 1.47 mM H_2_O_2_, 0.5 mM ascorbic acid (AsA), and 50 μM extracts. The assay reaction was beginning at the addition of H_2_O_2_, and the oxidation of ascorbic acid (AsA) was measured at 290 nm for 3 min. The activity of APX was expressed as μM min^-1^ mg^-1^ protein.

The GR activity was estimated through measuring the change of absorbance at 340 nm during 1 min due to the reduction in oxidized glutathione (Shaedle and Bassham, 1977), and glutathione reductase activity was expressed as nmol min^-1^ mg^-1^ protein.

### RNA Isolation, cDNA Synthesis, and PCR Assay

Frozen root, stem, and mature leaf samples of black locust were ground in liquid nitrogen. Total RNA was extracted with Trizol RNA isolation kit (Sangon, Shanghai, China) according to the manufacturer’s protocol. RNA integrity was checked by gel electrophoresis in 1.0% agarose gels and quantitative determination was carried out by spectrophotometric analysis with a Nano Drop 2000 (Thermo Scientific, Pittsburgh, PA, USA). RNA purity was estimated by calculating the A_260_/A_280_ ratio. Total RNA was reverse-transcribed to cDNA using a PrimeScript^TM^ RT reagent kit with gDNA Eraser (TaKaRa, Dalian, China) (Wormuth et al., 2006).

Quantitative real-time polymerase chain reaction was conducted using the CFX96 RT-PCR Detection System (Bio-Rad, Hercules, CA, USA). The 20-μL PCR reaction contained: 0.5 μL of each primer (**Supplementary Table S1**), 1 μL of cDNA template, 8 μL of double-distilled H_2_O, and 10 μL of SYBR Premix Ex Taq II (TaKaRa). The PCR procedure was as follows: denaturation at 94°C for 4 min, then 20 cycles at 94°C for 45 s, 58 or 60°C for 25 s, and 72°C for 10 s, and finally extension at 72°C for 10 min (Chen et al., 2013).

For each gene, the experiment was repeated three times (biological replicates), each biological replicate was the mean of three technical replicates. The results of relative quantification were estimated by using the method of 2^-ΔΔCT^ as described by Livak and Schmittgen (2001), and the transciriptional levels for each gene were normalized using the reference gene, *18S* rRNA (Chen et al., 2013).

### Statistical Analyses

Experimental data were subjected to two-way analyses of variance (ANOVAs) in SPSS 17.0 software (SPSS, Inc., Chicago, IL, USA). The means of each treatment were compared by Duncan’s test (α = 0.05) in a two-way ANOVAs. Two-way ANOVAs were performed to evaluate the significance of DS, the AM fungal inoculation treatment and the interaction of drought × inoculated treatment.

## Results

### Mycorrhizal Colonization and Biomass Production

None of NM seedlings were colonized by *R. irregularis*, whereas the inoculated black locust seedlings were observed to form the typical mycorrhizal structures (**Supplementary Figures S1A–C**). The colonization rates of AM seedlings were 76.8% under WW and 69.5% under DS.

**FIGURE 1:**
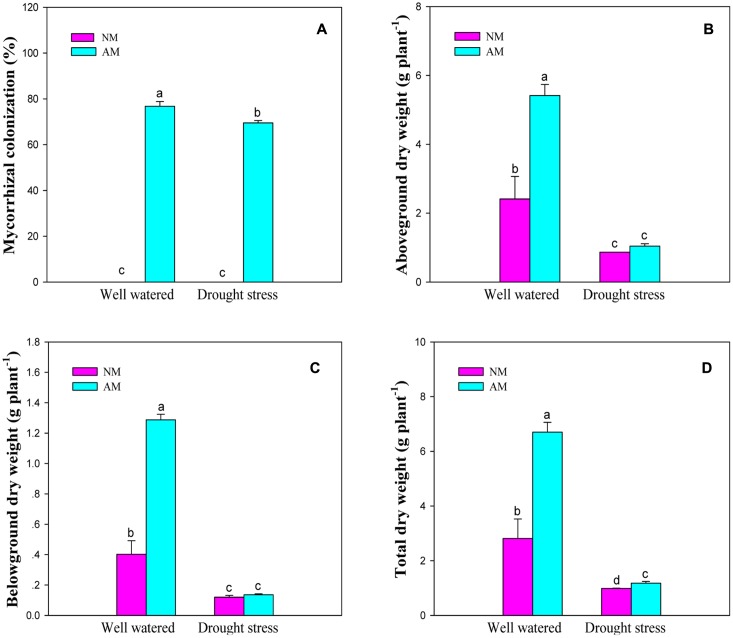
**Arbuscular mycorrhizal colonization and dry weight of black locust seedlings.** **(A)** Mycorrhizal colonization; **(B)** Aboveground dry weight; **(C)** Belowground dry weight; **(D)** Total dry weight. NM, non-mycorrhizal; AM, arbuscular mycorrhizal; WW, well-watered; DS, drought stress. Data are arithmetical mean ± SD (*n* = 6 biological replicates). Different lowercase letters (a, b, c, d) within each column denote significant differences at *P* ≤ 0.05.

Arbuscular mycorrhizal fungal inoculation, DS and their interaction significant (*P* ≤ 0.01) influenced plant growth traits such as aboveground, belowground, and total DW of black locust (**Supplementary Table S2**). DS produced a marked reduction in aboveground (64.0 and 80.6%), belowground (69.8 and 88.8%), and total (64.7 and 82.1%) DW of NM and AM seedlings, respectively. AM fungal inoculation increased aboveground, belowground and total DW under WW and DS conditions, relative to the NM treatment (**Figure 1**).

### Hydraulic Parameters

**FIGURE 2:**
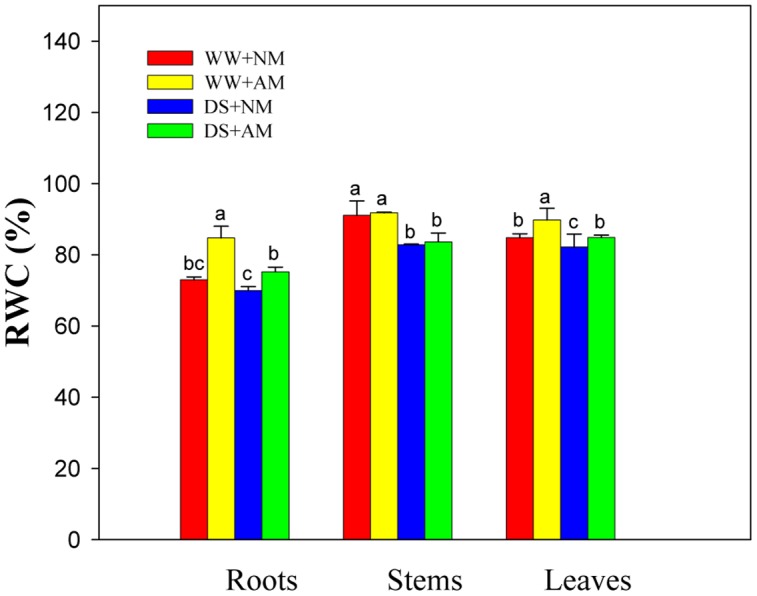
**Relative water content (RWC) in roots, stems, and leaves of black locust were affected by drought stress and AM fungal inoculation.** NM, non-mycorrhizal; AM, arbuscular mycorrhizal; WW, well-watered; DS, drought stress. Data are arithmetical mean ± SD (*n* = 6 biological replicates). Different lowercase letters in the column denote significant differences between treatments at *P* ≤ 0.05.

Drought stress significantly (*P* ≤ 0.01) affected RWC of leaves, stems, and roots of black locust seedlings. AM fungal inoculation significantly (*P* ≤ 0.01) influenced RWC of leaves and roots, but AM and DS emerged an interaction only on RWC of black locust roots (**Supplementary Table S2**). Under WW, AM fungal inoculation produced a marked increase in root (16.1%) and leaf (5.8%) RWC (*P* ≤ 0.05). Also, Mycorrhizal black locust also showed higher leaf (5.8%) and root (7.5%) RWC (*P* ≤ 0.05) under DS (**Figure 2**).

### Photosynthetic Pigment

**FIGURE 3:**
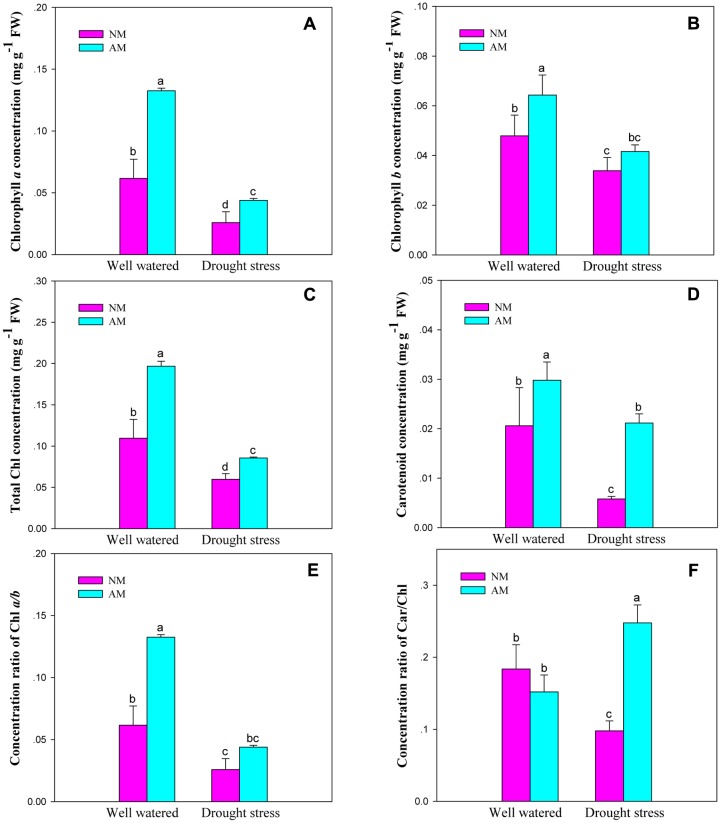
**Leaf concentrations of chlorophyll *a* (Chl *a*,** **A)**, chlorophyll *b* (Chl *b*, **B**), total chlorophyll (total Chl, **C**), carotenoid (Car, **D**), and the concentration ratios of chlorophyll *a/b* (Chl *a/b*, **E**) and carotenoid/chlorophyll (Car/Chl, **F**) of black locust were affected by drought stress and AM fungal inoculation. NM, non-mycorrhizal; AM, arbuscular mycorrhizal; WW, well-watered; DS, drought stress. Data are arithmetical mean ± SD (*n* = 6 biological replicates). Different lowercase letters above the error bars denote significant differences at *P* ≤ 0.05.

Mycorrhizal fungal inoculation influenced Chl and Car concentrations, and the concentration ratios for Chl *a*/*b* and Car/Chl of black locust seedlings (*P* ≤ 0.05) (**Supplementary Table S2**). DS influenced all the six pigment parameters (*P* ≤ 0.01), but the Car/Chl is an exception. DS and AM displayed an interaction on Chl *a* and total Chl concentrations, as well as the Car/Chl (*P* ≤ 0.01). DS caused a large reduction in the investigated pigment parameters. However, AM fungal inoculation improved Chl and Car concentrations, and the ratio of Chl *a*/*b* and Car/Chl of black locust leaves under different watering regimes. The differences were significant between NM and AM plants except for Chl *b* and Chl *a*/*b* under DS and Car/Chl under WW (**Figure 3**).

### Chl Fluorescence Parameters

**FIGURE 4:**
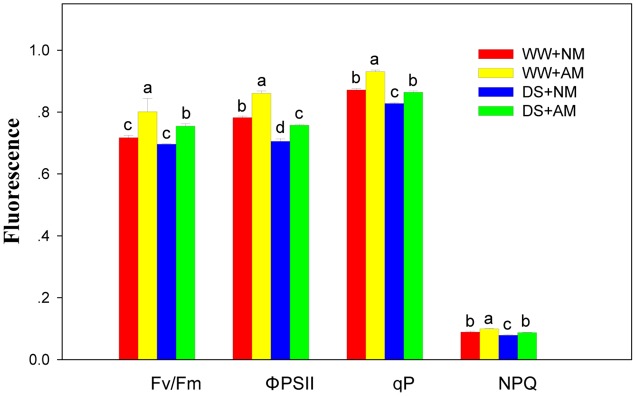
**Chl fluorescence parameters in black locust leaves were affected by drought stress and AM fungal inoculation.** NM, non-mycorrhizal; AM, arbuscular mycorrhizal; WW, well-watered; DS, drought stress. Data are arithmetical mean ± SD (*n* = 6 biological replicates). Different lowercase letters above the error bars denote significant differences at *P* ≤ 0.05.

Arbuscular mycorrhizal fungal inoculation significantly (*P* ≤ 0.01) influenced Chl fluorescence parameters of black locust seedlings, including q_p_, Φ_PSII_, F_v_/F_m_, and NPQ. DS significantly (*P* ≤ 0.01) influenced Chl fluorescence parameters, except for F_v_/F_m_. But all the Chl fluorescence parameters did not influenced by the interaction between AM and DS (**Supplementary Table S2**). Compared with NM black locust, the q_p_, Φ_PSII_, F_v_/F_m_, and NPQ of AM seedlings were 6.9, 10.3, 11.1, and 11.2% higher under WW, respectively; and 3.6, 7.5, 7.1, and 10.1% higher under DS, respectively. DS led to a decrease in Chl fluorescence parameters of black locust seedlings. Under DS, the q_p_, Φ_PSII_, F_v_/F_m_, and NPQ of NM seedlings decreased by 4.6, 9.8, 2.9, and 11.2% respectively, and by 7.5, 11.9, 5.9, and 12.1% respectively in AM seedlings (**Figure 4**).

### ROS Generation and Lipid Peroxidation

**FIGURE 5:**
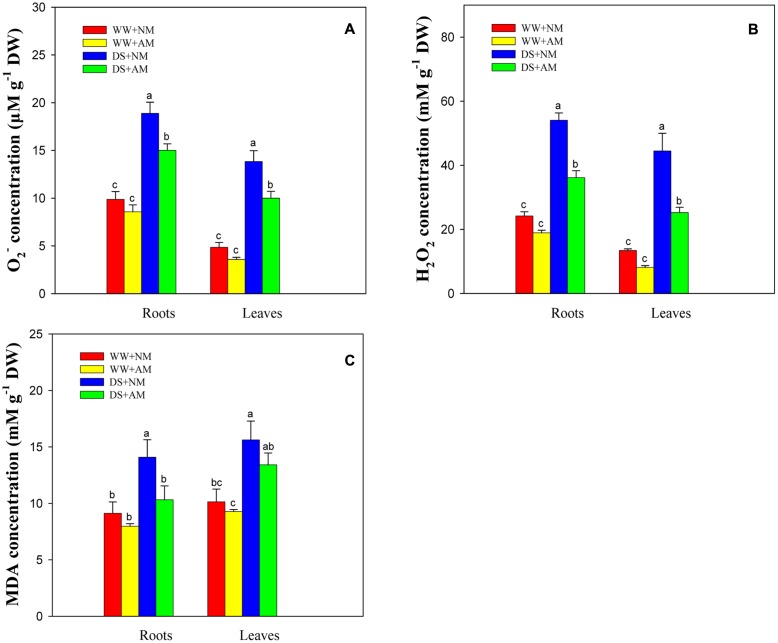
**Superoxide (O_2_^-^,** **A)**, hydrogen peroxide (H_2_O_2_, **B**) and malondialdehyde (MDA, **C**) concentrations in black locust leaves and roots were affected by drought stress and AM fungal inoculation. NM, non-mycorrhizal; AM, arbuscular mycorrhizal. Data are arithmetical mean ± SD (*n* = 6 biological replicates). Different lowercase letters above the error bars denote significant differences at *P* ≤ 0.05.

Drought stress significantly (*P* ≤ 0.01) influenced O_2_^-^, H_2_O_2_, and MDA concentrations of black locust seedlings. AM fungal inoculation significantly (*P* ≤ 0.05) influenced O_2_^-^ and H_2_O_2_ concentrations, and the interaction between AM and DS significantly (*P* ≤ 0.05) influenced H_2_O_2_ concentration (**Supplementary Table S2**). DS led to an increase in ROS generation and lipid peroxidation in black locust seedlings, but AM fungal inoculation treatment induced lower oxidative burst. Seedlings inoculated with AM fungi and grown under WW had less root and leaf O_2_^-^ (13.2 and 26.1%), H_2_O_2_ (21.7 and 39.5%), and MDA (12.6 and 8.5%) concentrations compared with NM seedlings. Under DS, the root and leaf O_2_^-^, H_2_O_2_, MDA concentrations of AM seedlings were 20.5 and 27.7%, 33.1 and 43.2%, 26.8 and 14.1% lower, respectively, than those of NM seedlings (**Figures 5A–C**).

### Antioxidant Enzyme Activity

**FIGURE 6:**
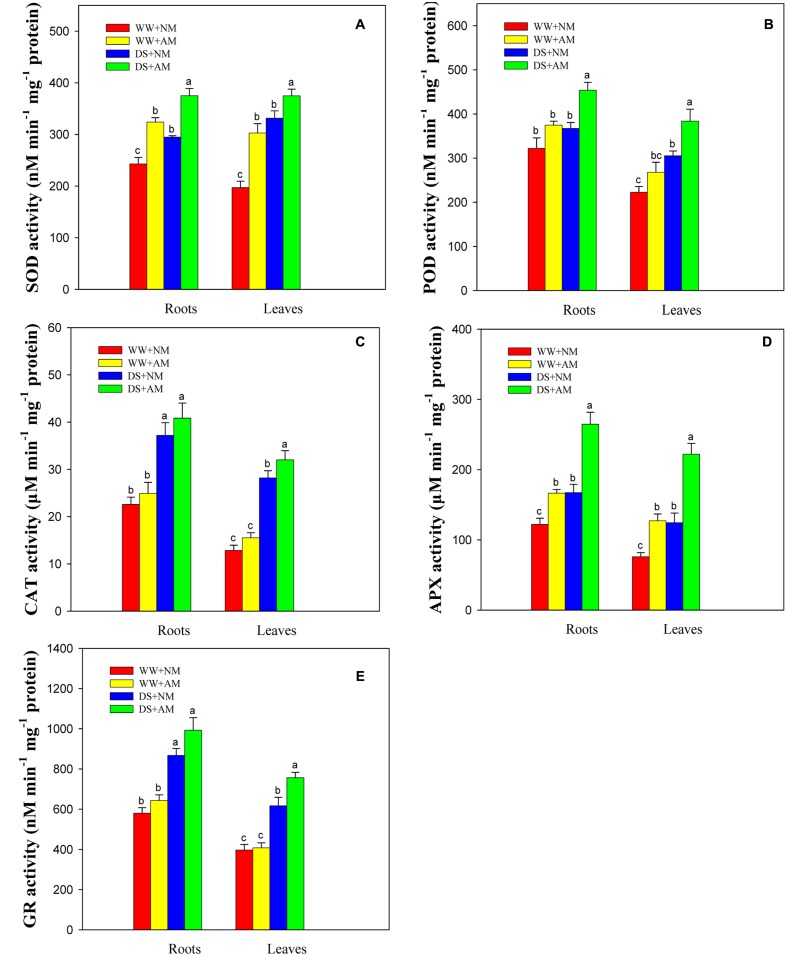
**Superoxide dismutase (SOD,** **A)**, peroxidase (POD, **B**), catalase (CAT, **C**), ascorbate peroxidase (APX, **D**), and glutathione reductase (GR, **E**) activities in black locust leaves and roots were affected by drought stress and AM fungal inoculation. NM, non-mycorrhizal; AM, arbuscular mycorrhizal. Data are arithmetical mean ± SD (*n* = 6 biological replicates). Different lowercase letters above the error bars denote significant differences at *P* ≤ 0.05.

Root and leaf SOD, POD, APX, GR activities were significantly (*P* ≤ 0.05) affected by the AM treatment and DS, but not by the interaction between AM and DS (*P* ≥ 0.05) (**Supplementary Table S2**). Antioxidant enzyme activities in both roots and leaves were significantly enhanced under DS, irrespective of AM status (**Figures 6A–E**). The root and leaf antioxidant enzyme activities of *R. irregularis*-inoculated seedlings were higher than those of NM seedlings under both water conditions. Seedlings inoculated with *R. irregularis* and grown under WW had higher root and leaf SOD (33.3 and 53.6%), POD (16.3 and 20.4%), CAT (10.3 and 20.9%), APX (36.4 and 67.4%), and GR (11.0 and 2.7%) activities compared with NM seedlings. Under DS, the root and leaf SOD, POD, CAT, APX, GR activities of AM seedlings were 27.2 and 13.1%, 23.5 and 25.7%, 9.8 and 13.5%, 58.2 and 78.3%, 14.4 and 22.7% higher than those of NM seedlings, respectively (**Figures 6A–E**).

### Antioxidant Gene Expression

Drought stress significantly (*P* ≤ 0.01) influenced expression levels of three antioxidant genes of black locust seedlings, and DS led to significant elevation in root, stem, and leaf expression of *Cu/Zn-SOD. APX*, and *GR* in both AM and NM plants compared with WW. AM fungal inoculation significantly (*P* ≤ 0.01) influenced gene expression levels of *Cu/Zn-SOD* (root, stem, and leaf), *APX* (stem and leaf), and *GR* (root). The interaction between AM and DS was significant (*P* ≤ 0.05) for gene expression levels of *Cu/Zn-SOD. APX*, and *GR*, except for *APX* (stem) (**Supplementary Table S2**).

**FIGURE 7:**
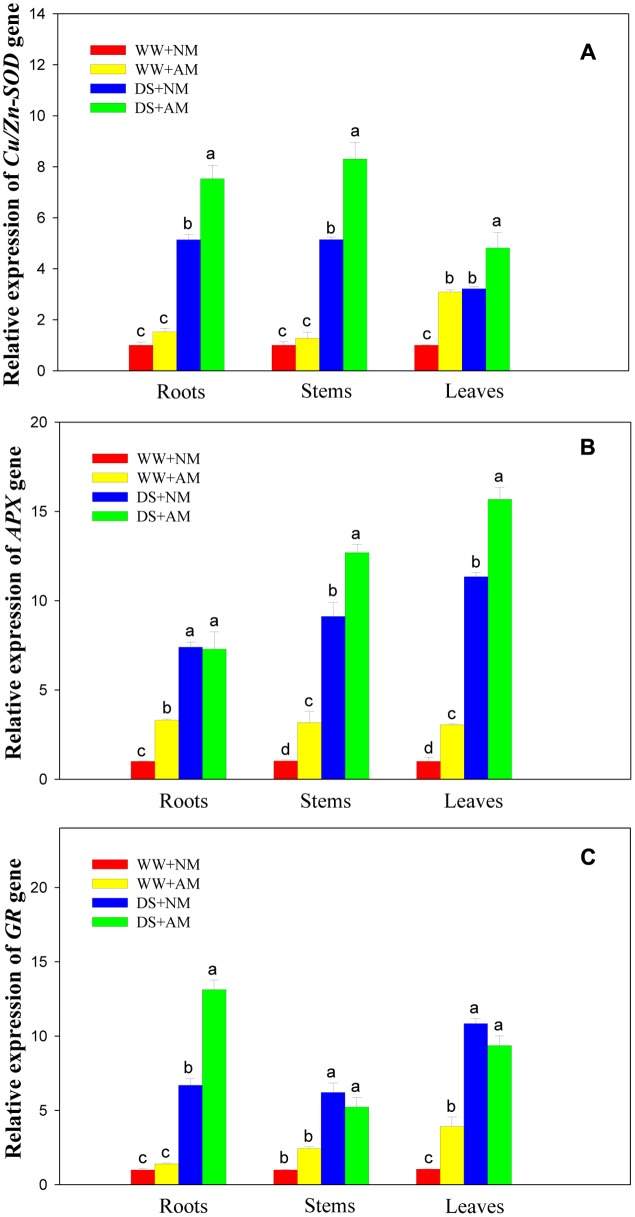
**Effects of *Rhizophagus irregularis* inoculation on *Cu/Zn-SOD. APX*, and *GR* gene expression in black locust seedlings exposed to moderate drought stress.** **(A)** Relative expression of *Cu/Zn-SOD* gene; **(B)** Relative expression of *APX* gene; **(C)** Relative expression of *GR* gene. WW, well-watered; DS, drought stress; NM, non-mycorrhizal; AM, arbuscular mycorrhizal. Data are arithmetical mean ± SD (*n* = 3 biological replicates). Relative gene expression in roots, stems, and leaves was examined after 14 days of drought stress. Relative quantification was assessed using the 2^-ΔΔCT^ method, with *18S rRNA* as the reference gene.

Root and stem expression levels of *Cu/Zn-SOD* showed no significant difference between NM and AM seedlings under WW. But leaf expression level of *Cu/Zn-SOD* in AM seedlings were more than threefold those in NM seedlings. Under DS, root, stem, and leaf expression levels of *Cu/Zn-SOD* in AM seedlings were 1.5-, 1.6-, and 1.5-fold those in NM seedlings (**Figure 7A**).

Under WW, root, stem, and leaf expression levels of *APX* in AM seedlings were 3.3-, 3.2-, and 3.1-fold those in NM seedlings. Under DS, root expression level of *APX* showed no significant difference between NM and AM seedlings, but stem and leaf expression levels of *APX* were 39.1 and 38.1% higher in AM than those in NM seedlings (**Figure 7B**).

Under WW, root and stem expression levels of *GR* showed no significant difference between NM and AM seedlings, but leaf expression levels of *GR* in AM seedlings was 3.9-fold those in NM seedlings. Under DS, root expression of *GR* in AM seedlings was close to twofold that in NM seedlings, while no significant differences were observed in stem and leaf expression levels of *GR* between NM and AM black locust seedlings (**Figure 7C**).

## Discussion

### Drought Inhibited Mycorrhizal Colonization, But Promoted RWC and Biomass of Black Locust

Previous studies in trifoliate orange (*Poncirus trifoliata*) (Zou et al., 2014), and black locust (Yang et al., 2014) have reported that mycorrhizal colonization was strongly inhibited by DS. This study confirms that drought restricted mycorrhizal root colonization of black locust by *R. irregularis*, mainly because water deficit inhibited the germination of spores and the growth of mycelia in the rhizosphere soil of host plant, or limited the transmission of infection from spores (Estaun, 1990). Although mycorrhizal infection reduced under drought, the improvement of plant growth in AM seedlings was still found. Colonized by *R. irregularis* remarkably enhanced the aboveground, belowground and the total DW of black locust, irrespective of the soil watering regimes. The results are in accordance with that of Yang et al. (2014) who observed aboveground and belowground DW increased in *Funneliformis mosseae*-colonized and *Rhizophagus intraradices*-colonized black locust seedlings as compared to NM plants under DS and well-watered conditions. Increase in plant DW by AM symbiosis probably related to the increasement of water absorption through AM fungal mycelia (Smith and Smith, 2011). *R. irregularis* inoculation of black locust seedlings promoted the absorption of water as revealed by the significantly higher RWC, an indicator of the water status in plants, in roots and leaves of AM black locust compared with those of non-AM plants under different watering regimes. However, detailed information about the relations among AM fungi and the metabolic pathways of water in the tissues of black locust are still poorly understood, which needs further research.

### Photosynthetic Response of AM to Drought Stress

Our results from the present study confirm the beneficial effect of AM fungal inoculation on chlorophyll concentration of black locust. *Glomus intraradices* inoculation of black locust enhanced the concentrations of pigments as revealed by the greatly higher Chl *a* and carotenoid, playing an important role in the process of plant photosynthesis (El-Qudah, 2014), in leaves of AM plants compared with NM seedlings under different soil water status. Nevertheless, the increasement of Chl concentration in mycorrhizal black locust has not been firmly linked to drought tolerance. The light energy, absorbed by Chl molecules in plant leaves, is used for photosynthetic electron transport, or is lost through regulating thermal dissipation, or is reemitted as Chl fluorescence, depending on the environmental and biochemical conditions (Liu et al., 2015). Chl fluorescence is used to reveal the photosynthetic efficiency which was denoted by values of maximum efficiency of PSII photochemistry and effective PSII quantum yield, and analyze the photosynthesis-related mechanisms that plant has suffered abiotic and biotic stress (Liu et al., 2015; Kong et al., 2016). Chl fluorescence parameter F_v_/F_m_ is used to detect stress in plants and reveal the maximum quantum yield of PSII which was indicated by ratios of variable to maximum fluorescence after dark-adaptation (Ibaraki and Murakami, 2006). Our results support previous findings reveal that AM symbiosis enhanced the F_v_/F_m_ in black locust leaves (Yang et al., 2015). We observed that the *R. irregularis* inoculation enhanced the drought resistance of black locust by reducing the decrease of F_v_/F_m_ and the photochemical quenching (q_p_) caused by drought. And mycorrhizal plants had higher F_v_/F_m_, Φ_PSII_, q_p_ and NPQ than non-mycorrhizal black locust, indicating that AM symbiosis has the ability to alleviate the adverse effect of DS on the PSII reaction center by improving the maximum quantum yield of PSII and photosynthetic capacity of black locust.

### Lower Accumulation of ROS for AM in Response to Drought Stress

It is well-documented that AM symbiosis is beneficial for the host plants to resist DS by an improvement of antioxidant system and a reduction of ROS (Zou et al., 2014). Here, the water deficit induced the accumulation of H_2_O_2_ and O_2_^-^ in black locust seedlings, irrespective of AM status. However, AM fungal inoculation decreased ROS generation in plant tissues under different watering regimes. The lower accumulation of ROS in AM seedlings might be involved in the intensive growth of hyphae and arbuscules within host roots (Fester and Hause, 2005).

H_2_O_2_, a major signaling substance of ROS, can spread to various parts of the cell and remain for a long time (Wu et al., 2007). It is well-documented that H_2_O_2_ could accumulate in intercellular fungal hyphae and clumped or less branched arbuscules in mycorrhizal plant roots (Salzer et al., 1999; Fester and Hause, 2005). Therefore, higher H_2_O_2_ accumulation in AM fungi may partly explain the lower levels of ROS in AM-colonized black locust seedlings, regardless of soil water status.

Malondialdehyde substantially reflects the extent of damage to cell membrane system caused by oxidative stress (Anjum et al., 2012). Various abiotic stresses such as drought, salt, extreme temperature result in overproduction of ROS causing oxidative damage to lipid in plants (Sharma et al., 2012; Fouad et al., 2014). We observed that DS notably increased MDA concentrations in different tissues of black locust, which is consistent with a previous research in olive plant (Fouad et al., 2014). Colonization by *R. irregularis* reduced MDA concentration in black locust seedlings under well-watered and drought conditions. The reduction in MDA can be attributed to the regulation of antioxidant protective and osmotic adjustment system (Wu et al., 2013b). MDA has been considered as an important reflection of weakening plant tolerance to abiotic stresses (Sharma et al., 2012; Fouad et al., 2014). Thus, lower MDA in mycorrhizal seedlings may reflect better plant growth under DS, therefore increasing plant tolerance to water deficit.

### Antioxidant Gene-Enzymes Response of AM to Drought Stress

Soil drought generally results in the disruption of cellular homeostasis, excess production of ROS, and oxidative damage to plant cell tissues, but plants possess a series of antioxidant enzymes (e.g., SOD, POD, CAT, APX, and GR) to control the level of ROS and protect against oxidative injury (Liu et al., 2011; Sharma et al., 2012). Data presented herein showed that mycorrhizal induced increases in SOD, POD, and CAT activities under drought and well-watered conditions, which is in agreement with the finding of Fouad et al. (2014) in olive plants. Our result indicates that *R. irregularis* inoculation induce an enhancement of certain antioxidant enzyme activities as a protective mechanism to scavenge excess ROS in black locust.

In black locust, the total SOD activity showed an increase in *R. irregularis*-colonized seedlings compared with NM treatments. The results are consistent with a study that observed an increase of SOD activity as a result of AM symbiosis (Ruiz-Lozano et al., 1996). Whereas, Cu/Zn-SOD is probably present in both the AM fungus (González-Guerrero et al., 2010) and the host plant, which is responsible for the enhanced SOD activity (Ruiz-Lozano et al., 1996) in AM plant species. Approving the result, Gralla and Valentine (1991) found that Cu/Zn-SOD in *Saccharomyces cerevisiae*, which accounts for more than 90% of the total SOD activity.

In the last few decades, the isoenzymatic pattern of SOD was studied in the symbiosis of *F. mosseae* and *Trifolium pratense*. Cu/Zn-SOD was observed in the *F. mosseae* spore, whereas Mn-SOD1, Cu/Zn-SOD1, and Cu/Zn-SOD2 were found in both leaves and roots of *T. pratense*. While mycorrhizal plant roots exhibited two kinds of new isozyme, Mn-SOD2 and mycCu/Zn-SOD that were specific isozymes respond to infection by AM fungus, probably due to an increase in the production of O_2_^-^ in the roots of host plant (Palma et al., 1993). Furthermore, cDNAs encoding SODs displayed distinct expression profiles in response to AM and drought (Ruiz-Lozano et al., 2001). Hence, there is speculation that a higher SOD activity in mycorrhizal black locust seedlings might result from a combination of specific isozymes, and the expression of genes encoding SODs.

As expected, a pronounced increase was observed in *Cu/Zn-SOD* gene expression as a consequence of mycorrhizal formation, regardless of watering regimes. The expression pattern can likely be attributed to the regulation of intracellular colonization of the host plant by AM-forming fungi (Salzer et al., 1999). In the germinated spores of an AM fungus, *Gigaspora margarita*, a functional *Cu/Zn-SOD* gene might offer protection as a ROS-inactivating system against localized host defense responses raised in arbuscule-containing cells (Lanfranco et al., 2005).

No significant associations were observed between root SOD/POD activity and root O_2_^-^/H_2_O_2_ concentration (*P* > 0.05), indicating that lower levels of ROS in AM seedlings may not be entirely attributed to the increased SOD and POD activities by mycorrhization. Actually, excess ROS can be scavenged by other antioxidant enzymes such as GR and APX. These two antioxidant enzymes operate in the ascorbate–glutathione cycle, a major pathway responsible for detoxification of H_2_O_2_ in green plant chloroplasts (Kang et al., 2013). Our results revealed that mycorrhizal induced much higher APX and GR activities under drought, showing a consistent effect of AM fungi in alleviation of oxidative stress. The substantially higher antioxidant enzyme activities, together with lower H_2_O_2_ accumulation and less lipid peroxidation, could explain the much less oxidative stress in AM seedlings. This result gives a proof that AM fungi protect black locust against oxidative stress, in turn enhancing drought resistance in the host plant (Fouad et al., 2014).

In our study, we observed that drought enhanced the expression of *APX* and *GR* in NM and AM seedlings of black locust, which is consistent with the findings of Furlan et al. (2014) in peanut. For *APX*, these variations might be associated with increased APX activity, whereas APX activities did not exactly go along with the changes of *APX* transcript levels. A similar result has been reported by Bian and Jiang (2009) in Kentucky bluegrass. Yet the expression of *APX* was up-regulated at the transcription and protein levels in pea subjected to DS, as described by Mittler and Zilinskas (1994). For *APX*, the discrepancy between gene expression and enzyme activity suggested that increased APX activity was not completely correlated with *APX* transcript levels, but perhaps regulated at the post-transcriptional level.

Certainly, further research is needed to understand the relations among AM fungi and the metabolic pathways of SOD, POD, CAT, APX, and GR in black locust. Additionally, it is still essential to recognize that non-enzymatic antioxidants or/and other antioxidant enzymes might be involved and should be studied next.

## Conclusion

**FIGURE 8:**
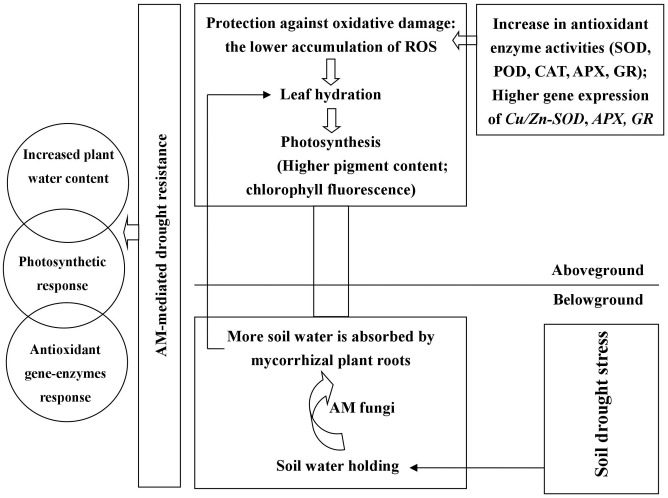
**Roles of AM fungal inoculation in black locust resistance to drought stress.** AM fungal inoculation enhanced RWC of root and promoted root-to-leaf transportation. Mycorrhizal black locust had higher gene expression levels of *Cu/Zn-SOD. APX* and *GR*, as well as SOD, POD, CAT, APX, and GR activities but lower O_2_^-^, H_2_O_2_, MDA concentrations compared with non-mycorrhizal plants. AM fungal inoculation increased chlorophyll concentrations and promoted photosynthesis under drought stress. AM fungal colonization causes physiological changes in aboveground parts probably through secreting glomalin, regulating endogenous hormones, stress-related proteins, and/or signaling molecules.

The symbiosis between black locust and AM fungus (*R. irregularis*) was well-established under DS and well-watered conditions (**Figure 1**). *R. irregularis* inoculation could increase RWC of tissues and thus protect plants from leaf wilting. Mycorrhizal black locust seedlings showed lower oxidative stress, but higher photosynthetic pigment concentration, photochemistry efficiency, as well as activity and gene expression of antioxidant enzymes than non-mycorrhizal plants, regardless of watering regimes, indicating that the AM fungus *R. irregularis* can confer a great degree of drought tolerance to the host plant black locust. The former protects the latter against oxidative stress by increasing antioxidant enzyme activities and gene expression that are responsible for elimination of excess ROS. A model is presented in **Figure 8**, which summarizing the roles of AM fungal inoculation in black locust resistance to DS. The effects of AM fungal inoculation on physiological changes of plant aboveground parts are probably attributed to the secretion of glomalin, the regulation of endogenous hormones, stress-related proteins, and/or signaling molecules, which requires to be further studied. Certainly, further researches also need to be conducted to elucidate the relations among AM fungi and the metabolic pathways of antioxidant enzymes, and the specific functions of each antioxidant gene regulated by AM symbiosis, in order to reveal the mechanism of AM symbiosis to alter plant drought adaptability.

## Author Contributions

Experimental design by FH and MT. Experimental execution, data analysis and paper written by FH. Contributed materials by MS and MT.

## Conflict of InterestStatement

The authors declare that the research was conducted in the absence of any commercial or financial relationships that could be construed as a potential conflict of interest.

## Abbreviations

AM: arbuscular mycorrhizal
ANOVA: analysis of variance
APX: ascorbate peroxidase
*APX*: ascorbate peroxidase gene
CAT: catalase
cDNA: complementary DNA
*Cu/Zn-SOD*: Cu/Zn-superoxide dismutase gene
DS: drought stress
GR: glutathione reductase
*GR*: glutathione reductase gene
H_2_O_2_: hydrogen peroxide
KI: potassium iodide
KOH: potassium hydroxide
MDA: malondialdehyde
NaClO: sodium hypochlorite
NM: non-inoculation
O_2_^-^: superoxide
PCR: polymerase chain reaction
POD: peroxidase
qRT-PCR: quantitative real-time PCR
rRNA: ribosomal RNA
RNA: ribonucleic acid
ROS: reactive oxygen species
SOD: superoxide dismutase
SPSS: statistic package for social science
TCA: trichloroacetic acid
WW: well-watered
