# Supplementary material for: Effects of Rhizophagus irregularis on Photosynthesis and Antioxidative Enzymatic System in Robinia pseudoacacia L. under Drought Stress
Source: Front Plant Sci. 2017 Feb 16;8:183. doi: 10.3389/fpls.2017.00183 (PMC5311038; doi:10.3389/fpls.2017.00183)
Supplement: Supplementary file 1 [file Data_Sheet_1.docx]

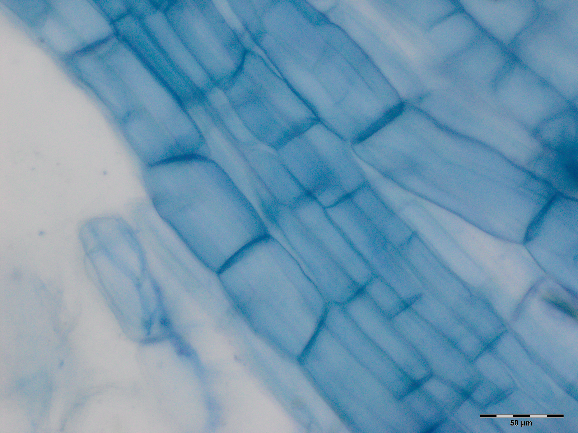


**A**


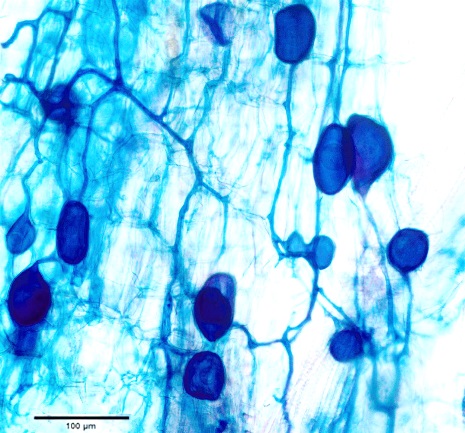


a

**B**


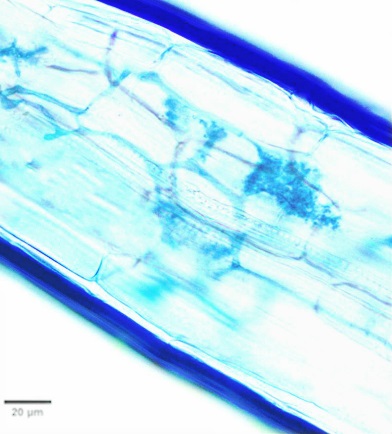


b

**C**

Fig. S1 Photomicrographs showing the structure of arbuscular mycorrhizal fungus (*Rhizophagus irregularis*) in roots of black locust (B, C). A, root cells of the no *Rhizophagus irregularis* inoculation treatment; B and C, arbuscular mycorrhizal fungi structures in the *Rhizophagus irregularis* inoculation treatment roots. a, vesicle; b. arbuscule. Scale bars: A=50 μm; B =100 μm; C = 20 μm.Table

Table S1 Primers used for quantitative real-time PCR in this study

| Name | | Nucleotide sequence (5'-3') | Product size (bp) | Annealing temperature (°C) |
| --- | --- | --- | --- | --- |
| *18S rRNA* | F | TAGTTGGTGGAGCGATTTGTC | 211 | 60 |
|  | R | CAGAACATCTAAGGGCATCACAG |  |  |
| *Cu/Zn-SOD* | F | CAACTGGACCACATTTCAA | 183 | 58 |
|  | R | GGACAACAACAGCCCTTC |  |  |
| *APX* | F | TCTGAGGGACGTGT | 160 | 60 |
|  | R | CTCCGTGAAGTATGAGTTGT |  |  |
| *GR* | F | CCACTATCTCCTCCGAAAC | 176 | 60 |
|  | R | GCTATCAAACTACTCCCAATC |  |  |

Table S2 Effects of arbuscular mycorrhizal fungal inoculation (AM), drought stress (DS), and AM×DS on plant parameters of black locust seedlings

| Parameters | Tissue | AM | | | DS | | | AM×DS | | |
| --- | --- | --- | --- | --- | --- | --- | --- | --- | --- | --- |
|  |  | Mean square | *F* | *P* | Mean square | *F* | *P* | Mean square | *F* | *P* |
| Mycorrhizal colonization | Roots | 16044.8 | 12057.7 | ^**^ | 39.6 | 29.7 | ^**^ | 39.6 | 29.7 | ^**^ |
| Aboveground dry weight | Leaves+stems | 7.6 | 56.7 | ^**^ | 26.3 | 196.5 | ^**^ | 6.0 | 44.8 | ^**^ |
| Belowground dry weight | Roots | 0.6 | 254.6 | ^**^ | 1.5 | 644.5 | ^**^ | 0.6 | 236.9 | ^**^ |
| Total dry weight | Roots+Leaves+stems | 12.5 | 78.0 | ^**^ | 40.6 | 253.3 | ^**^ | 10.2 | 63.9 | ^**^ |
| RWC | Roots | 217.4 | 77.6 | ^**^ | 118.2 | 42.2 | ^**^ | 31.6 | 11.3 | ^**^ |
|  | Stems | 1.7 | 4.3 | NS | 204.0 | 502.4 | ^**^ | 0.1 | 0.1 | NS |
|  | Leaves | 44.0 | 24.6 | ^**^ | 43.0 | 24.0 | ^**^ | 3.6 | 2.0 | NS |
| Chl *a* concentration | Leaves | 0.1 | 73.4 | ^**^ | 0.1 | 143.5 | ^**^ | 0.1 | 25.9 | ^**^ |
| Chl *b* concentration | Leaves | 0.1 | 10.4 | ^*^ | 0.1 | 24.1 | ^**^ | 10.4 | 1.3 | NS |
| Total Chl concentration | Leaves | 0.1 | 63.8 | ^**^ | 0.1 | 129.5 | ^**^ | 0.1 | 18.9 | ^**^ |
| Car concentration | Leaves | 0 | 23.8 | ^**^ | 0 | 21.6 | ^**^ | 2.7 | 1.5 | NS |
| Chl *a/b* | Leaves | 0.9 | 14.7 | ^**^ | 1.7 | 29.3 | ^**^ | 0.2 | 3.7 | NS |
| Car/Chl | Leaves | 0.1 | 16.8 | ^**^ | 15.3 | 0.1 | NS | 0.1 | 39.7 | ^**^ |
| q_p_ | Leaves | 0.1 | 152.4 | ^**^ | 0.1 | 204.9 | ^**^ | 0.1 | 8.9 | ^*^ |
| Φ_PSII_ | Leaves | 0.1 | 99.3 | ^**^ | 0.1 | 188.7 | ^**^ | 0.1 | 3.9 | NS |
| F_v_/F_m_ | Leaves | 0.1 | 10.7 | ^**^ | 0.1 | 2.5 | NS | 0 | 0.4 | NS |
| NPQ | Leaves | 0 | 16.7 | ^**^ | 0 | 23.1 | ^**^ | -0.1 | 0.1 | NS |
| [O_2_^.-^](http://www.baidu.com/link?url=rmOSGUD3itT8NCTThwpJEEnps73zSHrVQC5cPwJTCpKifDwwC84J55BME5nMKPgvnhcTUVmyo2hy1YTIfP1JhK) concentration | Leaves | 19.6 | 12.3 | ^**^ | 177.8 | 111.8 | ^**^ | 4.9 | 3.1 | NS |
|  | Roots | 17.0 | 6.7 | ^*^ | 161.6 | 63.2 | ^**^ | 4.0 | 1.6 | NS |
| H_2_O_2_ concentration | Leaves | 450.5 | 17.8 | ^**^ | 1743.3 | 68.9 | ^**^ | 145.4 | 5.7 | ^*^ |
|  | Roots | 401.7 | 44.2 | ^**^ | 1664.7 | 183.3 | ^**^ | 119.5 | 13.2 | ^**^ |
| MDA concentration | Leaves | 7.1 | 1.8 | NS | 69.3 | 18.0 | ^**^ | 1.3 | 0.3 | NS |
|  | Roots | 18.1 | 4.9 | NS | 40.1 | 10.8 | ^*^ | 5.1 | 1.4 | NS |
| SOD activity | Leaves | 16687.0 | 26.3 | ^**^ | 31981.5 | 50.3 | ^**^ | 2901.1 | 4.6 | NS |
|  | Roots | 19491.6 | 59.7 | ^**^ | 7939.6 | 24.3 | ^**^ | 0.4 | 0.1 | NS |
| POD activity | Leaves | 11488.6 | 10.0 | ^*^ | 29590.9 | 25.8 | ^**^ | 820.7 | 0.7 | NS |
|  | Roots | 14526.6 | 17.1 | ^**^ | 11606.1 | 13.6 | ^**^ | 859.4 | 1.0 | NS |
| CAT activity | Leaves | 31.6 | 5.0 | NS | 761.0 | 121.0 | ^**^ | 1.0 | 0.2 | NS |
|  | Roots | 26.8 | 1.4 | NS | 699.7 | 37.1 | ^**^ | 1.3 | 0.1 | NS |
| APX activity | Leaves | 16576.6 | 40.1 | ^**^ | 15402.4 | 37.2 | ^**^ | 1602.5 | 3.9 | NS |
|  | Roots | 15107.2 | 38.1 | ^**^ | 15413.3 | 38.9 | ^**^ | 2100.7 | 5.3 | ^*^ |
| GR activity | Leaves | 16992.1 | 5.8 | ^*^ | 242492.2 | 83.4 | ^**^ | 12514.1 | 4.3 | NS |
|  | Roots | 26740.6 | 5.5 | ^*^ | 304420.8 | 62.1 | ^**^ | 2834.0 | 0.6 | NS |
| Gene expression of *Cu/Zn-SOD* | Leaves | 10.2 | 33.7 | ^**^ | 11.7 | 38.7 | ^**^ | 0.2 | 0.6 | NS |
|  | Stems | 8.9 | 23.5 | ^**^ | 93.6 | 247.7 | ^**^ | 6.3 | 16.6 | ^**^ |
|  | Roots | 6.4 | 24.8 | ^**^ | 77.3 | 299.3 | ^**^ | 2.6 | 10.2 | ^*^ |
| Gene expression of *APX* | Leaves | 30.7 | 76.2 | ^**^ | 395.6 | 983.0 | ^**^ | 3.9 | 9.6 | ^*^ |
|  | Stems | 24.5 | 25.8 | ^**^ | 232.9 | 245.6 | ^**^ | 1.5 | 1.6 | NS |
|  | Roots | 3.6 | 4.8 | NS | 80.9 | 107.2 | ^**^ | 4.4 | 5.8 | ^*^ |
| Gene expression of *GR* | Leaves | 1.5 | 2.1 | NS | 174.3 | 251.4 | ^**^ | 14.2 | 20.4 | ^**^ |
|  | Stems | 0.2 | 0.3 | NS | 48.0 | 79.4 | ^**^ | 4.4 | 7.3 | ^*^ |
|  | Roots | 35.2 | 80.2 | ^**^ | 227.5 | 518.3 | ^**^ | 27.2 | 62.1 | ^**^ |

Note: AM: arbuscular mycorrhiza; DS: drought stress. ^*^: 0.01 < *P* ≤ 0.05; ^**:^ *P* ≤ 0.01; NS: not significant.
